# Assessing Quality of Life Among Senior-Year Dental Versus Non-Dental Healthcare Students in Saudi Arabia: A Cross-Sectional Study Using the WHOQOL-BREF

**DOI:** 10.3390/healthcare14142035

**Published:** 2026-07-08

**Authors:** Nada J. Farsi, Hana Jamjoom, Omair Abughazalah, Ahmed Alzhrani

**Affiliations:** 1 Department of Dental Public Health, King Abdulaziz University, P.O. Box 80200, Jeddah 21589, Saudi Arabia; 2 Department of Conservative Dental Science, King Abdulaziz University, P.O. Box 80200, Jeddah 21589, Saudi Arabia; hjamjoom@kau.edu.sa; 3 Faculty of Dentistry, King Abdulaziz University, P.O. Box 80200, Jeddah 21589, Saudi Arabia; dr.omaer@gmail.com (O.A.); a1h4m1d8@gmail.com (A.A.)

**Keywords:** quality of life, dental, pharmacy, student, WHOQOL-BREF

## Abstract

Purpose: The well-being of dental students is crucial, yet it can be influenced by various factors, including academic stress and clinical practice. In this study, we aimed to assess the association of dental education with the quality of life (QoL) of dental students compared with the QoL of non-dental healthcare students, while also evaluating the QoL of all students across psychological, physical, social, and environmental domains. Materials and Methods: In this cross-sectional study conducted at King Abdulaziz University between December 2023 and March 2024, all undergraduate senior-year students from the faculties of dentistry, medicine, and pharmacy were surveyed with the World Health Organization Quality of Life scale (WHOQOL-BREF). Results: Of the 477 participants (372 men and 105 women), 174 were from the dental faculty, 152 from the faculty of pharmacy, and 151 from the faculty of medicine. The academic distribution comprised 32.9% fourth-year students, 30.6% fifth-year students, 12.8% sixth-year students, and 23.7% interns. Dental and non-dental students predominantly rated their QoL as “good” or “very good,” with the percentage of dental students who did so being slightly higher (73.0%) than that of non-dental students (71.6%). In contrast, more non-dental students reported higher satisfaction with health than dental students did (74.3% vs. 65.0%, respectively). Overall, dental students had lower QoL scores (mean 64.8 ± 14.1) than students in other health specialties did. Notably, no statistical difference was observed between student categories in the physical domain, whereas students in other health specialties demonstrated higher QoL scores than dental students did in the psychological, social, and environmental domains. Conclusions: This study underscores disparities in QoL among students in different health specialties, with dental students exhibiting lower scores in three domains. These findings emphasize the need for tailored interventions in dental education to address stressors and enhance mental health support.

## 1. Introduction

Quality of life (QoL) is a multidimensional concept used to explore various aspects of people’s lives, encompassing physical and psychological well-being, financial independence, social relationships, personal beliefs, and living conditions [1]. The World Health Organization (WHO) defines QoL as an individual’s perception of their position in life, influenced by the cultural and value systems they live within, as well as their goals, expectations, standards, and concerns (WHO Division of Mental Health). The WHO Quality of Life scale (WHOQOL-BREF) is a widely used tool for assessing QoL across diverse populations. The tool is valued for its brevity, reliability, and ability to capture nuanced aspects of well-being within different cultural contexts. The WHOQOL-BREF instrument assesses QoL through four primary domains: physical health, psychological health, social relationships, and environment. Each of these domains plays a crucial role in shaping the overall well-being of students. By integrating this tool in this study, we aimed to gain deeper insights into the multifaceted nature of students’ QoL, facilitating the identification of key factors that contribute to their overall well-being and informing interventions to enhance their academic and personal experiences.

QoL among health professionals, including dental students, is a significant concern for educators and researchers due to the high levels of stress, anxiety, and burnout reported in these fields [2,3,4]. Research indicates that dental students experience more stress and anxiety than other medical students do [5]. Cross-sectional studies reveal that over 10% of dentists suffer from burnout, a long-term consequence of occupational stress [6,7]. Dentists at high risk of burnout exhibit poorer health and engage in unhealthier behaviors than do their less stressed peers [8]. The physical and mental stress negatively influences the QoL of dentists and their general health [9].

Several factors contribute to the high stress and burnout levels among dental students, including heavy workloads, dealing with anxious or difficult patients, poor working conditions, causing pain in patients, handling medical emergencies, and the routine nature of the job [10,11]. These stressors can lead to academic difficulties, can negatively affect the QoL of dental students [12,13], and have adverse sequelae on student academic performance, producing a negative impact on overall QoL [3,14]. When comparing dental and medical students, Guse et al. [15] found that dental students had higher levels of stress, which could be due to the higher clinical and practical demands of their academic courses.

Numerous studies have shown that university students, in particular those in health sciences, have a lower QoL than the general population does [16,17]. In a study in Turkey, investigators found that undergraduate dental students had acceptable QoL in the physical, environmental, and social relationship domains, but lower scores in the psychological domain, with a positive correlation between QoL and health satisfaction [18]. In a WHOQOL-BREF survey of pharmaceutical undergraduates in northern Nigeria, they reported their QoL as fair [19]. In South Canara, India, however, most dentists rated their QoL as very good [20]. Similarly, in another study that used the WHOQOL-BREF, the investigators reported that 73% of medical students rated their QoL as very good or good, with 59% satisfied or very satisfied with their health [21].

The QoL of university students has been widely studied, but health sciences students experience more challenges because of their clinical exposure and academic demands. Dental students in particular report higher levels of stress and burnout than their peers in other health fields do, possibly due to the intensive clinical load and required technical skills. Such stressors can affect student well-being and academic performance. However, findings from studies that have compared dental students’ QoL to that of other healthcare students are conflicting.

Comparative studies on the QoL of dental students versus other medical students are also scarce, particularly within the Saudi Arabian context. Most existing studies focus on a single discipline or compare only two groups [22,23], leaving a gap in understanding how QoL differs across multiple healthcare disciplines within the same cultural and institutional setting. Furthermore, senior-year students represent an important population as they face the highest academic and clinical demands yet remain understudied. This study therefore aims to address this gap by providing a multi-discipline comparison among senior-year dental, medical, and pharmacy students at a Saudi Arabian university. Saudi Arabia also presents a unique context for such investigation, given its rapidly expanding healthcare education sector, the cultural and societal expectations placed on students, and the limited data comparing QoL across different health disciplines within the region. Evaluating the QoL of dental students may provide valuable insights into their perceptions of life, health, and career satisfaction. In this study, we aimed to examine differences in QoL between dental students and their peers in other healthcare fields.

## 2. Material and Methods

### 2.1. Study Design

This was a cross-sectional study conducted between December 2023 and March 2024.

### 2.2. Study Population

This cross-sectional study was conducted at King Abdulaziz University (faculties of dentistry, medicine, and pharmacy), Jeddah, Saudi Arabia. Eligible participants included all dental, medical, and pharmacy students in their senior year.

A census sampling strategy was used in which all eligible students were invited to participate through official university emails, WhatsApp groups managed by class leaders, and in-class announcements. As all eligible senior-year students were invited to participate, a formal sample size calculation was not required.

### 2.3. Measurement Instrument

The English version of the WHOQOL-BREF questionnaire was used to compare the QoL of dental students to that of medical and pharmacy students. This questionnaire has reported similar reliability and internal consistency to that of the longer [24] 100-item version [25,26]. The WHOQOL-BREF consists of 26 items: the first two assess overall QoL and satisfaction with health, while the remaining 24 items cover four domains: (1) physical health (seven items), (2) psychological health (six items), (3) social relationships (three items), and (4) environment (eight items). For cultural reasons specific to the Saudi Arabian context, two items (Q11 and Q21) were excluded. Item Q11 asks about satisfaction with one’s sex life, and Q21 asks about satisfaction with one’s personal relationships in a romantic context; both were deemed culturally inappropriate for an unmarried, predominantly conservative student population. This practice is consistent with previous studies conducted in similar cultural contexts that have also omitted these items from the WHOQOL-BREF without compromising the overall validity of the instrument [23]. The internal consistency of the adapted domains was confirmed with acceptable to good Cronbach’s alpha values: physical domain α = 0.659, psychological domain α = 0.719, social domain α = 0.732, and environmental domain α = 0.749. Additional demographic questions were added to capture gender, age, marital status, faculty, and academic year.

### 2.4. Questionnaire Distribution

The questionnaire was digitalized by using the Google form, and the link was distributed to all health science students through university emails, WhatsApp groups through the leaders of each group, and class announcements. Paper copies of the questionnaire were also handed to the leader of each year. Several methods were used to distribute the questionnaire to increase participation in the study.

Participation was voluntary and anonymous. Participants were informed about the study aim at the beginning of both the online and paper versions of the survey. Participants were informed that completion and submission of the questionnaire, whether online via Google Forms or in hard copy, was considered as implicit consent to participate in the study. Completed hard copy questionnaires were returned in sealed envelopes to ensure confidentiality.

### 2.5. Scoring System

QoL scores were calculated in accordance with the WHOQOL-BREF manual (WHO user manual 1998). First, the negatively phrased items, Q3, Q4, and Q26, were coded reversely to ensure that higher values consistently indicated a more positive response and a higher QoL. This meant that a response of 1 became 5, 2 became 4, 3 remained the same, 4 became 2, and 5 became 1. Every response in the four domains was scored by using a 5-point Likert scale. The scoring was done in a positive direction: the higher the positive score, the higher the QoL. Next, we computed the domain scores. Each domain score was created by taking the mean of the items within that domain and multiplying it by 4. This calculation allowed the scores to be directly comparable to those obtained from the longer WHOQOL-100. We also calculated an overall score, combining all domains. Finally, the domain scores were converted to a 0–100 scale to make it easier to interpret. This conversion was done by subtracting 4 from each domain score, which is the lowest possible item score of 1 on a 1–5 scale, and then standardizing the result on a 100-point scale, using the formula (score−4) × (100/16). This method standardized the domain scores on a standardized scale. One hundred represented the best QoL, and zero represented the worst, according to the WHO manual recommendation for scoring.

### 2.6. Statistical Analyses

Sociodemographic characteristics of the participants are presented as descriptive statistics, continuous variables as means and standard deviations, and categorical variables as counts and percentages.

The global ratings of QoL and health satisfaction between students from the faculty of dentistry and those from other health science specialties were compared by using Fisher’s exact test. The frequency and percentage distribution of WHOQOL-BREF item responses are presented as counts and percentages. For ease of interpretation, the item responses were recategorized into three category variables. Depending on the item responses, for some items, “not at all” and “small amount” were placed in one category; “moderate amount” was placed in a separate category; and “a great deal” and “extreme amount” were placed in a third category. For other items, “very dissatisfied” and “fairly dissatisfied” were placed in one category, “neither satisfied nor dissatisfied” was placed in another category, and “satisfied” and “very satisfied” were placed in the last category.

The normality of each domain score and the overall score were assessed by conducting skewness and kurtosis tests, as well as by visually inspecting the histograms of the scores. Both tests indicated deviations from normality in the scores; therefore, non-parametric tests were used.

The WHOQOL-BREF scores for each domain and the overall scale are presented as mean, standard deviation, median, and interquartile range. The scores between dental students and those in other health specialties were compared by using the Wilcoxon rank-sum test. Comparisons across the three faculties were performed using the Kruskal–Wallis test. Pairwise post hoc comparisons were conducted using the Wilcoxon rank-sum test with Bonferroni correction for multiple comparisons.

Multivariable linear regression analyses were performed for each QoL domain (overall, physical, psychological, social, and environmental) to examine the independent association of faculty affiliation with QoL after adjusting for potential confounders including sex, age, academic year, marital status, and presence of medical illness.

*p*-values ≤ 0.05 were considered statistically significant. All statistical analyses were conducted by using Stata version 12.1 software (StataCorp LP, College Station, TX, USA).

### 2.7. Ethical Approval

This study received ethical approval (238-11-23) (4579296) from the Research Ethics Committee in the Faculty of Dentistry at King Abdulaziz University, Saudi Arabia.

## 3. Results

A total of 477 students participated in the study, with men representing 78% of the sample. The mean age of participants was 23 ± 1.5 years and most were single (467, 97.9%) rather than married (10, 2.1%). Among the participants, 174 (36.5%) were dental students, 152 (31.9%) were from the Faculty of Pharmacy, and 151 (31.7%) were from the faculty of medicine. The academic year included 157 (32.9%) students in fourth year, 146 (30.6%) students in fifth year, 61 (12.8%) students in sixth year, and 113 (23.7%) interns (Table 1).

A comparison of the global ratings between dentistry students and other health specialty students is shown in Table 2. Most students in both groups rated their QoL as “good” or “very good,” a slightly higher proportion of dental students than non-dental students doing so in these two categories combined (73.0% vs. 71.6%, respectively). Similarly, for health satisfaction, most students from both groups felt “satisfied” or “very satisfied,” with a greater percentage of non-dental students (74.3%) than dental students (65.0%) being in these categories. These differences in the ratings were not statistically significant (*p* = 0.874 for QoL rating; *p* = 0.098 for health satisfaction rating).

As shown in Table 3, 18% of participants reported that physical pain significantly hinders their ability to perform necessary tasks. Regarding the need for medical treatment in daily life, 12.8% of students indicated a moderate to extreme requirement. Although 65% of respondents expressed a high degree of life enjoyment, 70% perceived their lives as meaningful to a great or extreme extent. A high percentage, 74.6%, felt safe in their daily environments. In addition, 53.9% had sufficient time and opportunities for leisure activities, and 69.8% had effective communication skills for social interactions. This helped 57.9% of students to feel satisfied with their ability to perform daily activities and their work capacity. However, only 43% reported being satisfied or very satisfied with their sleep. Nevertheless, over 71.6% of students were highly satisfied with their living conditions, health services, and transportation. More than 60% of students were content with themselves, their personal relationships, and the support from friends. Conversely, 42.1% of students sometimes experienced negative feelings, such as despair and anxiety.

A comparison of WHOQOL-BREF scores across various domains, as well as a comparison of the overall scale between dental students and those in other health specialties, is presented in Table 4. Dentistry students reported a lower overall QoL score (mean 64.8 ± 14.1) than did students in other health specialties (mean of 69.2 ± 12.9, *p*= 0.001). In the physical domain, no significant difference was observed between groups of students (*p* = 0.477). However, students in other health specialties demonstrated significantly higher QoL scores than dental students in the psychological, social, and environmental domains.

The other health specialties were also assessed separately, as shown in Table 5. In the overall QoL, dental students scored significantly lower than medical students only (D vs. M, *p* < 0.05). No significant differences were found between any of the three faculties in the physical domain (*p* = 0.408). In the psychological domain, dental students scored significantly lower than both pharmacy and medical students (D vs. P and D vs. M, *p* < 0.05). In the social domain, dental students scored significantly lower than medical students (D vs. M, *p* < 0.05). In the environmental domain, medical students scored significantly higher than both dental and pharmacy students (D vs. M and P vs. M, *p* < 0.05).

In general, the study reveals differences in QoL domains among students of dentistry, pharmacy, and medicine. Dental students scored significantly lower than medical students in the overall, social, and environmental domains, and lower than both pharmacy and medical students in the psychological domain.

After adjusting for potential confounders including sex, age, academic year, marital status, and presence of medical illness, faculty affiliation was not an independent predictor of QoL in any domain (Table 6). The only variable that was significantly and consistently associated with lower QoL scores across all domains was the presence of a medical illness (overall: B = −7.30, 95% CI: −12.47, −2.14; physical: B = −7.71, 95% CI: −13.45, −1.97; psychological: B = −6.53, 95% CI: −12.92, −0.13; social: B = −9.14, 95% CI: −17.90, −0.38; environmental: B = −5.85, 95% CI: −11.30, −0.39). Older age was also independently associated with lower physical QoL scores (B = −1.48, 95% CI: −2.68, −0.28). Sex, marital status, and academic year were not significantly associated with QoL in any domain.

## 4. Discussion

This study delves into the QoL of students across various health specialties—dental, medical, and pharmacy—at King Abdul Aziz University in Saudi Arabia, in which we used the WHOQOL-BREF instrument to gauge their subjective well-being. Through the exploration of differences in QoL domains among these student cohorts, this study offers valuable insights into potential areas for targeted interventions to improve student welfare.

The demographic profile of the participants reflects a predominantly male, young, and single population, which is characteristic of students pursuing higher education in healthcare fields. Interestingly, despite variations in gender distribution and academic year across the three disciplines, we did not find statistically significant differences in global QoL ratings or health satisfaction levels between dental students and those in other health specialties. Although dental students ranked their overall QoL slightly higher than other students did, this was in agreement with a study by Cagri Burdurlu et al. [18]. Other studies on dental students in the Kingdom also ranked their QoL as good and satisfactory [22,23]. This finding suggests that factors that affect overall QoL and health satisfaction are likely influenced more by common educational stressors and experiences shared across health disciplines rather than discipline-specific factors. Within the Saudi Arabian context specifically, our findings are consistent with those of Al-Shibani and Al-Kattan, [23], and Alkallabi et al. [22], who reported satisfactory QoL among dental students in the Kingdom, and with Malibary et al. [21], who found that environmental health scored highest among medical students at King Abdulaziz University.

Dental students reported being satisfied with their health condition (65%), whereas medical and pharmacy students were highly satisfied (74%). This difference could be due to the dental profession being more demanding and patient commitment requiring more clinical competency, concentration, and hand dexterity. Being in the dental profession has been associated with musculoskeletal symptoms and stress, which may affect the QoL of the dentist, especially their psychological domain [27]. In China, Vo et al. [28] found that there is a direct correlation between the overall QoL and health satisfaction among Chinese dental students.

The multivariable regression analysis further revealed that after adjusting for demographic and health-related factors, faculty affiliation was no longer an independent predictor of QoL in any domain. This suggests that the observed differences between faculties may be partly explained by individual characteristics rather than discipline-specific factors alone. Notably, the presence of a medical illness emerged as the strongest independent predictor of lower QoL across all domains, underscoring the importance of health status in shaping students’ well-being regardless of their academic discipline.

The psychological domain had the lowest mean among dental and pharmacy students, while the physical domain scored lowest among medical students, in contrast to what has been reported by Nunes et al. [29] and Zeinabadi et al. [30], but in agreement with the results of Alrayes et al. [31]. The physical domain assesses the physical condition of a person and how it interferes with daily life activities. It also includes sleep and fatigue facets, energy, and the willingness of the person to perform everyday tasks. Healthcare students spend a large amount of time in clinics with long working hours. High physical demands are required to achieve patients’ well-being, predisposing dental students to experience musculoskeletal disorders [32,33,34]. University students are under constant pressure to perform and succeed in a highly competitive environment, which may result in poor sleeping habits and poor sleep quality related to stress [35], leading to low academic achievement [36]. These factors collectively contribute to diminished physical well-being, manifesting as low scores in the physical health domain on the WHOQOL-BREF instrument. In addition, significant differences were observed between pharmacy and medical students in environmental well-being, with medical students scoring highest, indicating varied experiences and stressors among students in different health disciplines. This finding underscores the importance of tailored approaches in dental education to address the unique challenges faced by dental students and to enhance their overall well-being.

Considering these findings, implementing targeted interventions such as stress reduction may help mitigate the psychological burden associated with dental education and promote student resilience. Furthermore, collaboration between dental, medical, and pharmacy faculties to share best practices in supporting student well-being could prove beneficial in creating a supportive learning environment conducive to the holistic development and well-being of all students in healthcare training programs.

Some limitations should be considered when interpreting our findings. As this was a cross-sectional study, we cannot establish causal relationships, and our findings reflect associations only. The study was conducted at a single university, which may affect generalizability. The sample was also predominantly male, which is reflective of the student population at our institution but may not represent other settings with a more balanced gender distribution. While we adjusted for several confounders in our regression analysis, other factors that could influence QoL, such as academic workload, mental health, social support, financial stress, and coping strategies, were not measured and should be explored in future studies. As with any self-reported questionnaire, reporting bias and social desirability bias cannot be ruled out.

## 5. Conclusions

This study sheds light on the QoL of dental, medical, and pharmacy students at King Abdulaziz University in Saudi Arabia in which we used the WHOQOL-BREF instrument to assess their subjective well-being across various domains. The findings reveal notable differences in QoL domains among students across different health specialties, with dental students demonstrating lower scores in the psychological, social, and environmental domains, while no significant difference was observed in the physical domain.

Despite variations in gender distribution and academic year in this study, we did not find statistically significant differences in global QoL ratings or health satisfaction levels between dental students and those in other health specialties. This finding highlights the importance of addressing the specific needs of students across different health specialties to enhance their overall QoL and well-being. By recognizing and addressing discipline-specific stressors and challenges, educators and administrators can foster a culture of support and resilience within healthcare training programs, ultimately promoting the success of future healthcare professionals and allowing them to flourish.

## Figures and Tables

**Table 1:** Sociodemographic Characteristics of the Respondents (*n* = 477).

Characteristic	*n* (%)
**Gender**	
Male	372 (78.0)
Female	105 (22.0)
Age, mean (SD)	23.0 (1.5)
**Marital status**	
Single	467 (97.9)
Married	10 (2.1)
**Faculty**	
Dentistry	174 (36.5)
Pharmacy	152 (31.9)
Medicine	151 (31.7)
**Year**	
Fourth	157 (32.9)
Fifth	146 (30.6)
Sixth	61 (12.8)
Intern	113 (23.7)
**Medical illness**	
Yes	27 (5.7)
No	450 (94.3)

Note: Values in table are shown as *n* (%) except where otherwise indicated.

**Table 2:** Comparative Global Ratings of Quality of Life and Health Between Dental and Other Health Sciences Students.

Global Rating Questions	Dentistry *n* (%)	Other Health Sciences *n* (%)	*p*-Value ^a^
**How would you rate your quality of life?**			
Very poor	2 (1.2)	2 (0.7)	0.874
Poor	7 (4.0)	13 (4.3)	
Neither poor nor good	38 (21.8)	71 (23.4)	
Good	93 (53.5)	150 (49.5)	
Very good	34 (19.5)	67 (22.1)	
**How satisfied are you with your health?**			
Very dissatisfied	4 (2.3)	3 (1.0)	0.098
Fairly dissatisfied	20 (11.5)	18 (5.9)	
Neither satisfied nor dissatisfied	37 (21.3)	57 (18.8)	
Satisfied	69 (39.7)	146 (48.2)	
Very satisfied	44 (25.3)	79 (26.1)	

Note: ^a^ Fisher’s exact test.

**Table 3:** Frequency and Percentage Distribution of WHOQOL-BREF Item Responses Among Health Sciences Students.

No.	Question	Not at All/Small Amount *n* (%)	Moderate Amount *n* (%)	A Great Deal/Extreme Amount *n* (%)
3	To what extent do you feel that physical pain prevents you from doing what you need to do? ^a^	301 (63.1)	90 (18.9)	86 (18.0)
4	How much do you need any medical treatment to function in your daily life? ^a^	357 (74.8)	59 (12.4)	61 (12.8)
5	How much do you enjoy life?	46 (9.6)	121 (25.4)	310 (65.0)
6	To what extent do you feel your life to be meaningful?	32 (6.7)	111 (23.3)	334 (70.0)
7	How well are you able to concentrate?	61 (13.4)	188 (41.1)	208 (45.5)
8	How safe do you feel in your daily life?	16 (3.5)	100 (21.9)	341 (74.6)
9	How healthy is your physical environment?	38 (8.3)	152 (33.3)	267 (58.4)
10	Do you have enough energy for everyday life?	74 (15.5)	169 (35.4)	234 (49.1)
12	Have you enough money to meet your needs?	83 (17.4)	151 (31.7)	243 (50.9)
13	How available to you is the information you need in your day-to-day life?	24 (5.0)	135 (28.3)	318 (66.7)
14	To what extent do you have the opportunity for leisure activities?	50 (10.5)	170 (35.6)	257 (53.9)
15	How well are you able to get around?	31 (6.5)	113 (23.7)	333 (69.8)
		**Very/Fairly Dissatisfied *n* (%)**	**Neither Satisfied nor Dissatisfied *n* (%)**	**Satisfied/Very Satisfied *n* (%)**
16	How satisfied are you with your sleep?	120 (25.2)	152 (31.9)	205 (43.0)
17	How satisfied are you with your ability to perform your daily living activities?	66 (13.8)	135 (28.3)	276 (57.9)
18	How satisfied are you with your capacity for work?	60 (12.6)	131 (27.4)	286 (60.0)
19	How satisfied are you with yourself?	58 (12.2)	106 (22.2)	313 (65.6)
20	How satisfied are you with your personal relationships?	46 (9.6)	135 (28.3)	296 (62.1)
22	How satisfied are you with the support you get from friends?	52 (10.9)	115 (24.1)	310 (65.0)
23	How satisfied are you with the conditions of your living place?	26 (5.5)	95 (19.9)	356 (74.6)
24	How satisfied are you with your access to health services?	30 (6.3)	95 (19.9)	352 (73.8)
25	How satisfied are you with your transport?	40 (8.4)	94 (19.7)	343 (71.9)
26	How often do you have negative feelings such as blue mood, despair, anxiety, depression? ^a^	147 (30.8)	201 (42.1)	129 (27.0)

Note: ^a^ Negatively worded statements have been accounted for in the analyses by reverting the responses. Abbreviation: WHOQOL-BREF, World Health Organization Quality of Life survey.

**Table 4:** Comparative Analysis of WHOQOL-BREF Scores Between Dentistry and Other Health Specialty Students.

Domain	Dentistry Mean (SD)	Dentistry Median (IQR)	Other Health Specialities Mean (SD)	Other Health Specialities Median (IQR)	*p*-Value *
Overall	64.8 (14.1)	65.9 (57.2, 74.2)	69.2 (12.9)	69.4 (61.3, 78.6)	0.001
Physical	65.4 (15.3)	67.9 (53.6, 75)	66.8 (14.5)	67.9 (57.1, 78.6)	0.477
Psychological	60.3 (17.7)	61.3 (50, 75)	66.6 (15.7)	70 (55, 75.0)	<0.001
Social	65.5 (24.1)	62.5 (50, 87.5)	70.9 (21.6)	75 (62.5, 87.5)	0.021
Environmental	68.1 (13.7)	68.8 (62.5, 75)	72.2 (14.4)	71.9 (65.6, 81.3)	0.001

Note: * Wilcoxon rank-sum test. Abbreviations: WHOQOL-BREF, World Health Organization Quality of Life survey; IQR, interquartile range.

**Table 5:** Comparative Analysis of WHOQOL-BREF Scores Among Dentistry, Medicine, and Pharmacy Students.

Domain	Dentistry (*n* = 174) Mean (SD)	Dentistry Median (IQR)	Pharmacy (*n* = 152) Mean (SD)	Pharmacy Median (IQR)	Medicine (*n* = 151) Mean (SD)	Medicine Median (IQR)	*p*-Value ^a^	Multiple Comparisons ^b^
Overall	64.8 (14.1)	65.9 (57.2, 74.1)	67.9 (12.8)	68.4 (60.5, 75.4)	70.4 (12.9)	70.8 (62.5, 80.4)	0.001	D vs. M *
Physical	65.4 (15.3)	67.9 (53.6, 75.0)	67.7 (15.3)	67.9 (57.1, 78.6)	65.9 (13.6)	64.3 (57.1, 75.0)	0.408	—
Psychological	60.3 (17.7)	61.2 (50.0, 73.8)	65.8 (15.7)	70.0 (55.0, 75.0)	67.4 (15.7)	70.0 (57.5, 80.0)	0.001	D vs. P *; D vs. M *
Social	65.5 (24.1)	62.5 (50.0, 87.5)	69.1 (19.8)	75.0 (50.0, 78.1)	72.8 (23.2)	75.0 (62.5, 93.8)	0.013	D vs. M *
Environmental	68.1 (13.7)	68.8 (62.5, 75.0)	68.9 (14.1)	68.8 (62.5, 75.8)	75.5 (14.0)	78.1 (68.8, 84.4)	<0.001	D vs. M *; P vs. M *

Abbreviations: D, dentistry students; M, medical students; P, pharmacy students; IQR, interquartile range; WHOQOL-BREF, World Health Organization Quality of Life survey. ^a^: Kruskal–Wallis test. ^b^: Bonferroni-adjusted pairwise Wilcoxon rank-sum tests. * indicates statistical significance (*p* < 0.05 after adjustment). — indicates no significant pairwise differences.

**Table 6:** Multivariable Linear Regression of WHOQOL-BREF Domain Scores (*n* = 477).

Predictor	Overall QoL B (95% CI)	Physical B (95% CI)	Psychological B (95% CI)	Social B (95% CI)	Environmental B (95% CI)
Pharmacy vs. Dentistry †	7.73 (−10.64, 26.11)	3.25 (−17.19, 23.69)	3.65 (−19.11, 26.42)	18.93 (−12.26, 50.13)	5.10 (−14.32, 24.51)
Medicine vs. Dentistry †	9.73 (−8.79, 28.25)	0.53 (−20.07, 21.13)	5.00 (−17.94, 27.94)	22.22 (−9.22, 53.66)	11.16 (−8.41, 30.73)
Female vs. Male †	−0.09 (−3.82, 3.65)	0.53 (−3.62, 4.69)	−2.75 (−7.37, 1.88)	−0.16 (−6.50, 6.18)	2.03 (−1.92, 5.97)
Age	−0.53 (−1.61, 0.55)	−1.48 (−2.68, −0.28) *	0.30 (−1.04, 1.63)	−0.30 (−2.13, 1.53)	−0.64 (−1.78, 0.49)
Married vs. Single †	−3.78 (−12.07, 4.51)	0.43 (−8.79, 9.66)	−8.50 (−18.77, 1.77)	−4.25 (−18.33, 9.82)	−2.79 (−11.55, 5.97)
Medical illness vs. None †	−7.30 (−12.47, −2.14) *	−7.71 (−13.45, −1.97) *	−6.53 (−12.92, −0.13) *	−9.14 (−17.90, −0.38) *	−5.85 (−11.30, −0.39) *
Year 5 vs. Year 4 †	−0.96 (−4.29, 2.37)	1.19 (−2.52, 4.89)	−1.62 (−5.75, 2.51)	−2.50 (−8.16, 3.16)	−0.91 (−4.43, 2.62)
Year 6 vs. Year 4 †	0.32 (−18.34, 18.97)	−0.58 (−21.33, 20.18)	−6.43 (−29.54, 16.68)	7.16 (−24.51, 38.83)	1.12 (−18.59, 20.83)
Intern vs. Year 4 †	7.55 (−10.91, 26.02)	5.38 (−15.16, 25.92)	1.05 (−21.83, 23.92)	18.87 (−12.48, 50.21)	4.92 (−14.59, 24.43)
Model Statistics					
R^2^	0.071	0.040	0.068	0.053	0.075
Adjusted R^2^	0.053	0.021	0.050	0.034	0.057
F *p*-value	0.0001	0.024	0.0001	0.003	<0.001

B = unstandardized regression coefficient; 95% CI = 95% confidence interval. * *p* < 0.05. † Reference categories: Dentistry, Male, Single, No medical illness, Year 4. Cronbach’s α for adapted domains: Physical 0.659; Psychological 0.719; Social 0.732; Environmental 0.749.

## Data Availability

The data that support the findings of this study are available from the corresponding author upon reasonable request.

## Abbreviations

IQR, interquartile range; QoL, quality of life; WHO, World Health Organization; WHOQOL-BREF, World Health Organization Quality of Life survey.
